# Fabrication of High-Strength and Porous Hybrid Scaffolds Based on Nano-Hydroxyapatite and Human-Like Collagen for Bone Tissue Regeneration

**DOI:** 10.3390/polym12010061

**Published:** 2020-01-01

**Authors:** Yannan Liu, Juan Gu, Daidi Fan

**Affiliations:** 1 Shanxi Key Laboratory of Degradable Biomedical Materials, School of Chemical Engineering, Northwest University, Xi′an 710069, China; liuyannan_2009@163.com (Y.L.); gu_juan89@163.com (J.G.); 2 Shanxi R&D Center of Biomaterials and Fermentation Engineering, School of Chemical Engineering, Northwest University, Xi′an 710069, China; 3 Biotech. & Biomed. Research Institute, Northwest University, Xi’an 710069, China

**Keywords:** scaffolds, bone tissue regeneration, biocompatibility, nano-hydroxyapatite, human-like collagen

## Abstract

A novel, three-dimensional, porous, human-like collagen (HLC)/nano-hydroxyapatite (n-HA) scaffold cross-linked by 1,2,7,8-diepoxyoctane (DEO) was successfully fabricated, which showed excellent mechanical and superior biological properties for bone tissue regeneration in this study. The physicochemical characterizations of different n-HA/HLC/DEO (nHD) scaffolds were investigated by determining the morphology, compression stress, elastic modulus, Young’s modulus and enzymatic hydrolysis behavior in vitro. The results demonstrated that nHD-2 and nHD-3 scaffolds showed superior mechanical properties and resistance to enzymatic hydrolysis compared to nHD-1 scaffolds. The cell viability, live cell staining and cell adhesion analysis results demonstrated that nHD-2 scaffolds exhibited low cytotoxicity and excellent cytocompatibility compared with nHD-1 and nHD-3 scaffolds. Furthermore, subcutaneous injections of nHD-2 scaffolds in rabbits produced superior anti-biodegradation effects and histocompatibility compared with injections of nHD-1 and nHD-3 scaffolds after 1, 2 and 4 weeks. In addition, the repair of bone defects in rabbits demonstrated that nHD-2 scaffolds presented an improved ability for guided bone regeneration and reconstruction compared to commercially available bone scaffold composite hydroxyapatite/collagen (HC). Collectively, the results show that nHD-2 scaffolds show promise for application in bone tissue engineering due to their excellent mechanical properties, anti-biodegradation, anti-biodegradation, biocompatibility and bone repair effects.

## 1. Introduction

Bone defects are frequently observed in patients with trauma, neoplasias, infection or corrective osteotomies. Clinically, it is necessary to perform bone grafting treatment, as large segmental bone defects cannot heal without treatment [1]. Normally, autologous bone grafts, allografts and xenografts serve as alternative materials to repair segmental bone defects [2]. Nevertheless, there are limitations of these bone grafting treatment approaches, such as restricted availability, poor mechanical properties, allograft rejection reactions and donor site morbidity [2]. Therefore, some new substitutes for damaged bone tissue reconstruction must be developed and applied, which has been a major challenge due to the complex implant circumstances and limited applicability of alternative devices [3,4].

To date, bone tissue engineering scaffolds have been demonstrated to be one of the most promising materials for bone defect repair [5,6]. Various natural biopolymers, synthetic biodegradable polymers and composite materials have been applied for the construction of porous scaffolds in bone tissue engineering [7,8]. Biologically, natural bone stroma is mainly composed of an inorganic component (collagen) and an organic component (bone apatite) [9]. Based on this, we designed compound scaffolds containing inorganic and organic phases to replace the individual material phase. For the inorganic phase, nano-hydroxyapatite (n-HA) has the advantages of good biocompatibility, high plasticity and remarkable mechanical properties, as its chemical and crystalline properties are analogous to those of bone apatite. Furthermore, it possesses an ultrafine structure and a high surface area, which is advantageous for cell-biomaterial interactions, and this has been extensively explored in bone engineering applications [10,11,12,13].

For the organic phase, collagen is a biodegradable material with excellent water retention ability and osteoinductivity that can be used as an artificial bone graft substitute [8,14]. However, the insufficient mechanical strength of collagen scaffolds restricts their direct application in vivo [15]. In this work, human-like collagen (HLC)—a hydrosoluble protein—was expressed by recombinant *Escherichia coli* from a human mRNA sequence [16]. As a prospective material, it has been successfully applied to artificial bone tissue scaffolds and vessel tissues due to its high water solubility, biodegradability, workability, and biocompatibility [17,18]. Hence, bone scaffolds combined with HLC and other macromolecules are promising for use in the tissue engineering field as scaffolds comprised of organic components for tissue repair.

Moreover, chemical crosslinking is the most frequently utilized scaffold technology to improve the biomechanical features and prevent the degradation of bone tissue engineering scaffolds through network bonding [5,6]. Recently, some chemical crosslinking agents have been applied to bone tissue regeneration, such as glutaraldehyde, carbodiimide (EDC) and genipin [5]. Glutaraldehyde has been widely used as a crosslinker to crosslink proteins and other macromolecules in various tissue scaffolds; however, free glutaraldehyde released during the biodegradation process of scaffolds would be deleterious in patients [19,20,21]. EDC has also been applied in bone tissue engineering as a result of its low cell toxicity, but EDC-crosslinked scaffolds have poor biomechanical properties [5,22]. Furthermore, genipin has often been investigated as a crosslinker and has been incrementally utilized in bone scaffolds because of its biocompatibility and low cytotoxicity, but the blue color of genipin-crosslinked scaffolds is usually not acceptable to patients [6,23]. In contrast, this report is introducing a new cross-linking agent, DEO, which is an epoxy compound [24]. It is colorless and possesses an epoxy group that reacts with human-like collagen to enhance the mechanical characteristics of crosslinked scaffolds. Additionally, residual free DEO is water soluble and can be readily removed by rinsing the crosslinked compounds, which does not cause harm to the animal [25,26].

In the present study, we focused on bone tissue regeneration scaffolds based on HLC and nHA crosslinked by DEO. The scaffolds were designated nHD-1, nHD-2 and nHD-3 in the light of the different concentrations of DEO used in them. The morphological characteristics, mechanical properties and in vitro degradation of the resulting scaffolds were investigated. The cellular compatibility of the scaffolds was analyzed through cell viability, cell attachment and live cell staining analyses. Furthermore, the biodegradability and histocompatibility of the scaffolds in vivo were studied via the subcutaneous injection of rabbits. Finally, the bone repair effect of the scaffolds was explored by using radius defects in rabbits as an experimental animal model.

## 2. Materials and Methods

### 2.1. Materials

HLC was expressed in *E. coli*, from a human mRNA sequence (China patent number: ZL01106757.8). n-HA (20 nm) was purchased from Epri Nano Materials Ltd. (Osaka, Japan). DEO was obtained from Sigma-Aldrich. Hydroxyapatite/collagen composite (HC) was obtained from the Beijing Yierkang Bioengineering Development Center (Beijing, China). Collagenase was obtained from Tokyo Chemical Industry Co. Ltd. (Tokyo, Japan). DMEM medium and fetal bovine serum were provided by HyClone (Logan, UT, USA). All other solvents and reagents were of analytical grade.

### 2.2. Fabrication of the nHD Scaffolds

To synthesize the nHD scaffolds, HLC was added to pyrogen-free water with magnetic stirring. Then, n-HA was slowly dispersed in HLC solution at an HLC/n-HA ratio of 3:1 (*w*/*w*) and was mixed thoroughly. The n-HA/HLC mixture was poured into a cylindroid mold that possessed twenty-four holes and was frozen by a programmable freezing instrument (kryo 550-16, Bath, Britain). To remove the water completely from the mixture and maintain the resulting three-dimensional porous structures, the mold was lyophilized with a vacuum freeze-dryer (SIM, Los Angeles, CA, USA) for 48 h under a vacuum of 6.7–13.3 Pa. The scaffolds were further crosslinked with DEO at concentrations of 4%, 6% and 8% (*v*/*v*) DEO, which were prepared in a 0.25 M NaOH aqueous solution at 50 °C for 48 h, and then the scaffolds were washed with ultrapure water to dislodge the crosslinker that did not participate in the reaction. To ensure the complete removal of the cross-linking agent, the scaffolds were washed with distilled water twice for 20 min each at 121 °C and used for the subsequent experiments. Finally, nHD-1, nHD-2 and nHD-3 scaffolds were obtained in the light of the concentrations of 4%, 6% and 8% (*v*/*v*) DEO, respectively.

### 2.3. Fourier-Transform Infrared Spectroscopy (FTIR)

The structures of the nHD scaffolds were determined using FTIR (Spectrum 2000, Perkin-Elmer, Ettlingen, Germany) by the potassium bromide pressed-disk method. FTIR spectra were collected from 4000 to 500 cm^−1^.

### 2.4. X-ray Diffractometry (XRD)

The crystalline phases of the nHD scaffolds were measured by XRD (Rigaku D/max-3C, Tokyo, Japan) at a working voltage of 40 kV, a rate of 2°/min and an angle range of 10–60°. The phases were investigated via comparison with the n-HA X-ray diffractograms.

### 2.5. Determination of Porosity

The porosities of the nHD scaffolds were measured by Archimedes′ Principle [27]. The porosity was determined using the following formula:

$$\mathrm{Porosity}=\frac{W_{t}-W_{r}-W_{d}}{W_{c}-W_{r}}\;\times 100\%,$$

where *W*_c_ is the weight of the container filled with ethanol, *W*_d_ is the dry weight of scaffold, *W*_t_ is the total weight of the container with scaffold following immersion of the scaffold in ethanol and *W*_r_ is the residual mass after removing scaffold. Three parallel samples for each group were measured.

### 2.6. Microscopic Investigation

The nHD scaffolds were expected to possess interconnected pores. Prior to the other experiments, the pole morphology of the scaffolds was investigated using scanning electron microscopy (SEM). The scaffolds were cut into small pieces, fixed on an aluminum plate, sprayed with platinum and observed under a SEM (Hitachi S-570, Tokyo, Japan). Furthermore, we randomly selected three replicate samples from the nHD-1, nHD-2 and nHD-3 scaffolds, respectively. Then the SEM images were obtained from each replicate samples, and the range of hole diameters of scaffolds in each group was measured using Photoshop software.

### 2.7. Mechanical Properties 

The scaffolds were modified into shapes of 10 mm in diameter and 20 mm in height. An INSTRON 5565 Materials Testing System (Boston, MA, USA) with a load of 5000 N was used to exert the maximum pressure that the samples could bear at a loading rate of 1 mm/min. Three parallel samples for each concentration were measured.

### 2.8. In Vitro Degradation

The in vitro degradation of the nHD scaffolds was investigated using collagenase (100 U/mL). The scaffolds were weighed, sterilized with Co-60 irradiation and immersed in tubes containing 2 mL of enzyme solution in a 37.0 °C incubator for 4 days. The weights of the scaffolds were recorded every day. The weight loss was calculated with the equation *W*_L_ = (*W*_0_ − *W*_1_)/*W*_0_ × 100%, where *W*_0_ and *W*_1_ are the weights of the scaffolds before and after enzymolysis, respectively. This process was performed in triplicate, and the mean value of the weight loss rate was computed.

### 2.9. Cell Culture and Viability Analysis

The cytotoxicity of the nHD scaffold extracts was evaluated in MC3T3-E1 osteoblast cells according to ISO standards (ISO10993.12-2004). The scaffolds were sterilized using Co-60 irradiation and placed into tubes with fresh culture medium at 37 °C for 24 h. Then, the leaching solution was reserved after the scaffolds were removed from the tubes. In this experiment, MC3T3-E1 cells were cultured on 96-well plates (100 μL/well) in a 37 °C incubator for 12 h. Afterwards, the culture medium was removed, and scaffold extract and fresh culture medium (blank group) were added to the different wells for 1, 3 and 5 days of incubation, respectively. After incubation, CCK-8 (Beyotime, Shanghai, China) reagent was added to each well, and the cells were incubated for 4 h. The absorbance was measured at 450 nm using a microplate reader (Bio-Rad, Hercules, CA, USA). The process was replicated in parallel three times.

### 2.10. Cell Attachment and Live Cell Staining Analysis

To observe the effects of the materials on the growth of MC3T3-E1 osteoblast cells, cells were evenly plated onto the scaffolds. Briefly, the scaffolds were sterilized by Co-60 irradiation and then cut into circular flakes of 10 mm diameter and 3 mm height, which were placed in 48-well culture plates. DMEM medium (1 mL/well) was added to the wells to prewet the scaffolds, which were incubated for 24 h in a CO_2_ (5%) incubator at 37 °C. The culture medium was removed, and cells were inoculated onto each film (50 μL/well) at a density of 1 × 10^5^ cells/mL. After the cells had adhered to the scaffolds for 3 h, 0.5 mL of an additional medium was added to each well, and the cells were cultured for 1, 3 and 5 days. Parts of the scaffolds were fixed in 2.5% glutaraldehyde, dehydrated in various concentrations of ethanol (30%, 50%, 70%, 90%, 95% and 100%), vacuum-dried and observed by SEM.

The other portions of the scaffolds were stained with acridine orange (AO) for 10 min to assess the cell growth on the scaffolds. The stained live cells were investigated using fluorescence microscopy. The experiment was replicated for three parallel samples.

### 2.11. Quantitative Analysis of Cell Adhesion

The cell proliferation on the nHD scaffolds was analyzed in quantitative via the CCK-8 assay. After the cells had adhered to the scaffolds for 1, 3 and 5 days, the cell-seeded scaffolds were removed into new 48-well plates and incubated using fresh culture medium including 10% CCK-8 for 4 h. The culture medium from each well (100 μL) was transferred to 96-well plates, and the optical density (OD) value was measured at 450 nm by a microplate reader. The process was replicated in parallel three times.

### 2.12. Subcutaneous Implantation Experiments

The in vivo biocompatibility of the scaffolds was evaluated through implanting the scaffolds into New Zealand rabbits. All animal experiments were carried out with the approval of the Northwest University Animal Ethics Committee.

Research-grade rabbits that were 3 months of age with body weights of 2 kg were selected. After shaving and disinfection, 1 cm-diameter nHD scaffolds were implanted subcutaneously into the backs of the rabbits. After 1, 2 and 4 weeks, the rabbits were killed by carbon dioxide inhalation, and the tissues containing skin and the materials were observed.

### 2.13. The Repair of Radius Defects

The 12 rabbits were all treated for two radius defects (right and left). In six of them, the nHD-2 scaffolds were implanted into the radius defect of the left leg, right radius defects were made without implantation as the blank group. In the other six rabbits, the positive control HC scaffolds were implanted into the radius defect of the left leg; right radius defects were also made in the rabbits in the blank group. The specific steps used for the operation were as follows: 3% sodium pentobarbital (1 mL/kg) was administered via auricular intravenous injection to anaesthetize the rabbits. The animal forearms were cleaned before shaving, and 5% iodine and 75% alcohol were used for the disinfection of the forearm. The periosteum was peeled from the radial forearm to reveal the middle part of the radius. A 17 mm bone defect was made with a dental bur in left and right radius of each rabbit, respectively. HC and nHD-2 were implanted into the radius defect site on the left legs of rabbits in different groups. The right legs with untreated radial defects of the rabbits were the blank groups. The deep fascia, subcutaneous tissue and skin were sutured layer by layer, and the incision was disinfected with 75% alcohol. A dressing was applied to the wound to bandage it and prevent it from moving. After the operation, each animal was housed alone in a cage, and 400,000 units of penicillin were injected intramuscularly for three days continuously. The radial defect and graft site were X-rayed on the day of the surgery and 4, 8 and 12 weeks after surgery by using the same conditions and scanning distance.

### 2.14. Histological Analysis

The 1 cm-thick tissues surrounding the radius defect repair site were fixed in 10% formaldehyde solution, decalcified, dehydrated, paraffin-embedded, serially sectioned and stained with hematoxylin and eosin. Then, the slices were observed with an optical microscope (Nikon Eclipse 80i, Tokyo, Japan).

### 2.15. Statistical Analysis

Data are presented as the means and standard deviations using GraphPad Prism 5 software (La Jolla, CA, USA). All statistical analyses were performed using SPSS software, version 16.0 (SPSS, Chicago, IL, USA); *p* < 0.05 was determined to be statistically significant.

## 3. Results and Discussion

### 3.1. Characterization of the nHD Scaffolds

**Figure 1:**
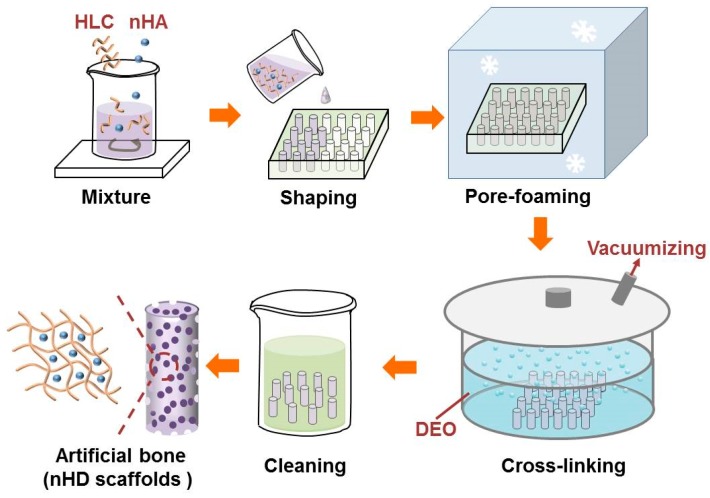
Schematic diagram of the synthesis of nano-hydroxyapatite (n-HA)/human-like collagen (HLC)/DEO (nHD) scaffolds.

**Figure 2:**
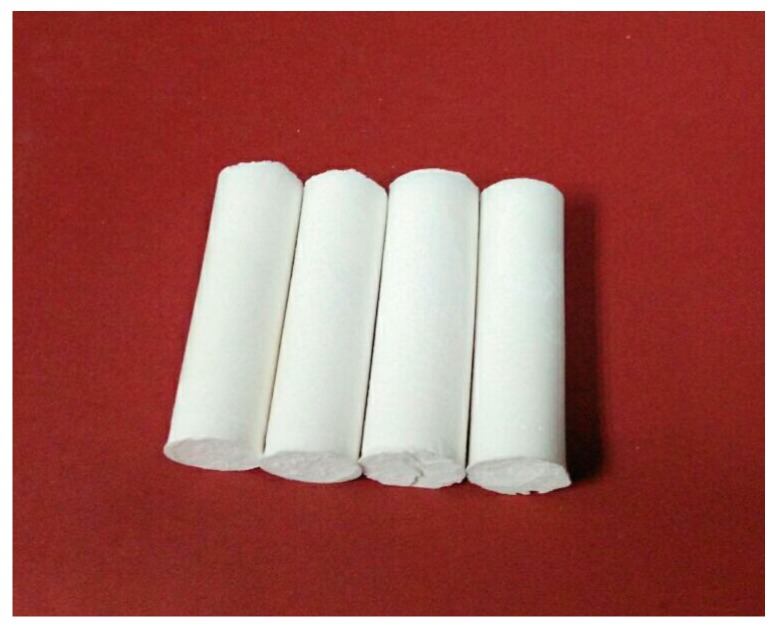
Macroscopic view of the nHD scaffolds.

**Figure 3:**
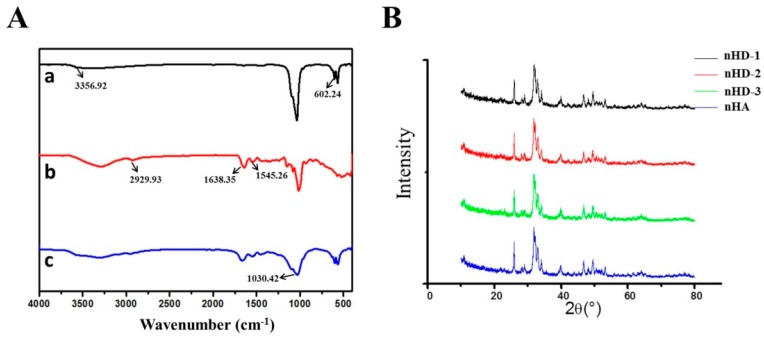
The characterization of nHD scaffolds. (**A**) FT-IR spectra of nHA (**a**), HLC (**b**) and nHD scaffolds (**c**). (**B**) XRD patterns for nHD-1, nHD-2 and nHD-3 scaffolds cross-linked by various concentrations of DEO, 4%, 6% and 8%, respectively.

A schematic of the synthesis of the nHD scaffolds is presented in Figure 1. The macroscopic view of the nHD scaffolds is indicated in Figure 2. The FT-IR spectra of the nHD scaffolds and individual n-HA and HLC materials were obtained (Figure 3A). The characteristic absorption peaks of nHA were 3356.92 and 602.24 cm^−1^, which represented the –OH stretching vibration peak and the PO_4_^3−^ peak, respectively. The characteristic absorption peaks of HLC were 2929.93, 1638.35 and 1545.26 cm^−1^, corresponding to the C–H peak, the C=O stretching vibration peak and the N–H bending vibration peak. In the spectra of the nHD scaffolds, a new characteristic peak associated with C–O–C bond appeared at 1030.42 cm^−1^ that was mainly due to the conjugation of the epoxide group in DEO and the carboxyl in HLC. These results indicated that we successfully synthesized nHD scaffolds via intermolecular interactions between HLC and DEO.

Figure 3B shows the X-ray diffraction patterns of the nHD-1, nHD-2 and nHD-3 scaffolds and nHA. The diffraction peaks of nHA were 26.1°, 32.3°, 33.0°, 34.2°, 40.1°, 46.9° and 49.9°, which are consistent with the standard XRD spectra of hydroxyapatite (JCPDS file number: 09-0432). Furthermore, these peaks are in compliance with the diffraction patterns of the nHD-1, nHD-2 and nHD-3 scaffolds. Collectively, the results demonstrated that the crystalline phase of the nHD scaffolds was calcium phosphate.

### 3.2. Mechanical Properties of the nHD Scaffolds

**Figure 4:**
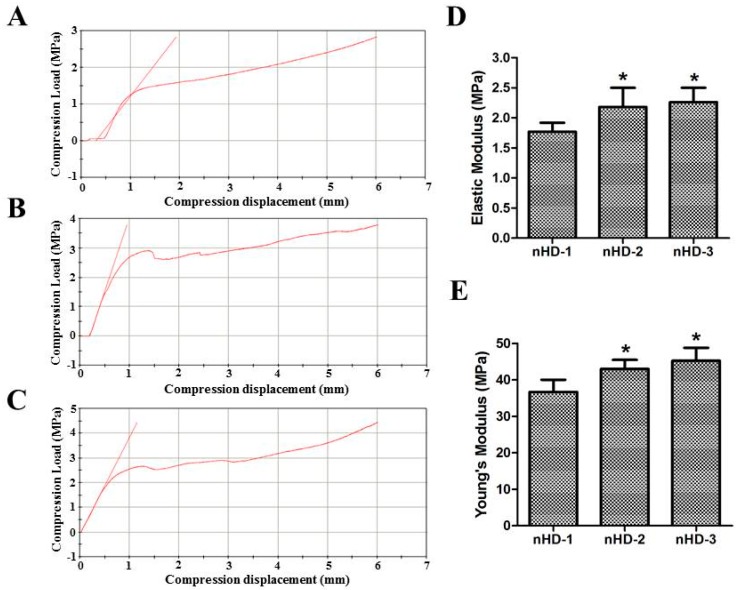
Mechanical properties of the three nHD scaffolds. (**A**–**C**) The compression loads of nHD-1 scaffolds (**A**), nHD-2 scaffolds (**B**) and nHD-3 scaffolds (**C**). (**D**) The elastic moduli of three nHD scaffolds. (**E**) The Young′s moduli of three nHD scaffolds; * *p* < 0.05 compared with nHD-1 scaffolds.

The mechanical properties of scaffolds are vital for bone tissue engineering, especially for hard tissue regeneration or for tissues that require initial strength in the primary period of regeneration process [28]. The mechanical performance of the scaffolds according to the compression stress, elastic modulus and Young′s modulus is shown in Figure 4A–E. The compression stresses of the nHD-1, nHD-2 and nHD-3 scaffolds with 6 mm of compression displacement reached 2.76, 3.87 and 4.26 MPa, respectively (Figure 4A–C). Furthermore, as indicated in Figure 4D, the elastic moduli of the nHD-1, nHD-2 and nHD-3 scaffolds were 1.77 ± 0.15, 2.18 ± 0.32 and 2.26 ± 0.24 MPa, respectively. Compared to nHD-1, the elastic moduli of the nHD-2 and nHD-3 scaffolds both showed a significant increase (*p* < 0.05; Figure 4D). Figure 4E shows that the Young′s moduli of nHD-1, nHD-2 and nHD-3 were 39.68 ± 3.35, 46.01 ± 2.48 and 48.23 ± 3.59 MPa, respectively. Compared with that of nHD-1, the Young’s moduli of the nHD-2 and nHD-3 scaffolds all showed obvious increases (*p* < 0.05). The increase in the DEO concentration enhanced the mechanical strength of the nHD scaffolds in a dose-dependent manner. The mechanical characteristics of the scaffolds play an important role in matching to the tissue specificity of the extracellular matrix [29]. The ideal scaffold of bone tissue engineering must have excellent mechanical strength to provide support for new tissue [30]. Previous studies have indicated that the compression stress and Young′s modulus of nHA composite scaffolds used for bone tissue engineering are 2.97 and 43.03 MPa, respectively [31]. Moreover, many investigations have shown that the compression stress of collagen-based scaffolds used as bone tissue materials is lower than 3.0 MPa [8,17,32]. In contrast, our nHD scaffolds showed significantly enhanced mechanical properties. Overall, nHD scaffolds generated with a higher concentration of crosslinking agent had a superior capacity for resistance to deformation.

### 3.3. Structure of the Scaffolds before and after Enzymatic Degradation In Vitro

**Figure 5:**
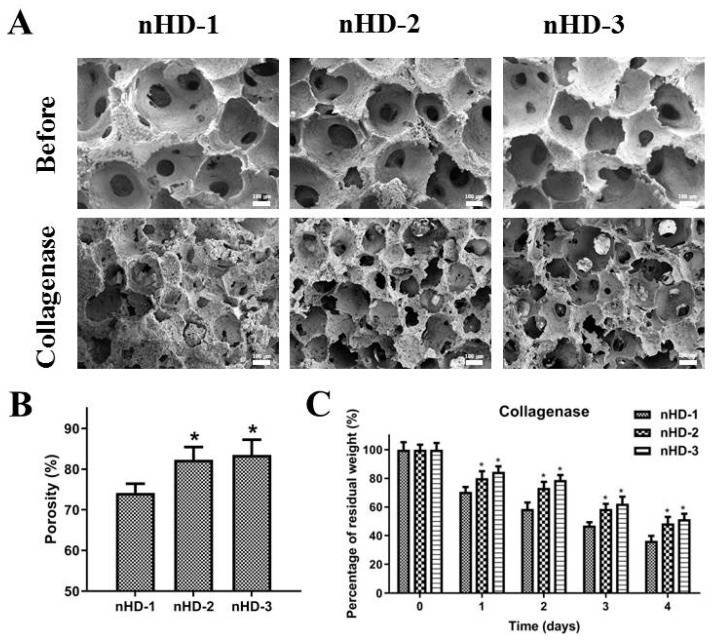
(**A**) SEM micrographs of the three nHD scaffolds at 37 °C before and after degradation by collagenase; the scale bar is 100 μm. (**B**) The porosity of the three nHD scaffolds. (**C**) The percentage of residual weight for the three nHD scaffolds in vitro collagenase degradation test; * *p* < 0.05 compared with nHD-1 scaffolds.

Highly interconnected porous networks of materials can provide an environment suitable for nutrient transportation and cell growth and proliferation [33]. As shown in Figure 5A, nHD-1, nHD-2 and nHD-3 scaffolds all possessed evenly porous structures and good interpenetrating network structures. The hole diameters of the three scaffolds were approximately 150 to 360 µm, which is beneficial for cell growth and nutrient exchange. The smaller average pore size (<100 µm) can interfere with cell distribution and restricts cell migration to the center of scaffolds [34,35]. On the contrary, if the pore size is too large, this may affect the mechanical property of scaffolds and the production of extracellular matrix on scaffolds [35,36]. Hence, the pore sizes of the nHD scaffolds were suitable for bone tissue engineering application, which were in accordance with previous studies about bone regeneration scaffolds [34,37]. Furthermore, the porosities of the composite scaffolds are also crucial elements for fabricating suitable scaffolds for bone tissue regeneration [34]. As shown in Figure 5B, the porosities of the nHD-1, nHD-2 and nHD-3 scaffolds were 75.2% ± 2.21%, 82.3% ± 3.15% and 83.5% ± 3.78%, respectively. Compared to nHD-1, the porosities of the nHD-2 and nHD-3 scaffolds both showed an obvious increase (*p*<0.05). The high porosity of the scaffolds has a significant effect on the biological applicability which provides a larger surface area for cellular migration, proliferation, gaseous exchange and nutrient transport [13,38]. Previous studies showed that the porosity of collagen composite scaffolds used for bone tissue engineering were less than 80% [27,31,36]. In comparison, our nHD-2 and nHD-3 scaffolds exhibited higher porosities and are more suitable for bone regeneration application. 

To explore the effect of the DEO concentration in the nHD scaffolds on degradation, we performed enzymatic degradation studies in vitro. The inner morphologies of the nHD scaffolds after 4 days of collagenase degradation at 37 °C are displayed in Figure 5A. After 4 days of degradation, the nHD-2 and nHD-3 scaffolds maintained the integrity and morphology of their three-dimensional architectures compared to those observed before degradation. However, the nHD-1 scaffolds exhibited disordered pores and showed the collapse of the substrate structure. Moreover, the residual weights of the scaffolds after 1, 2, 3 and 4 days of collagenase degradation were also explored. As shown in Figure 5C, the percentage of residual weight observed in the nHD-1, nHD-2 and nHD-3 scaffolds after 4 days of degradation were approximately 36.4% ± 3.4%, 48.5% ± 4.6% and 51.5% ± 3.8%, respectively. It was demonstrated that as the concentration of the crosslinking agent was increased, the percentage of residual weight of the nHD scaffolds increased. Collectively, the results showed that the high percentage of residual weight and the structural integrity of the nHD-2 and nHD-3 scaffolds enabled them to serve as suitable materials for application in the field of bone tissue engineering.

### 3.4. Scaffold Cytotoxicity Assay

**Figure 6:**
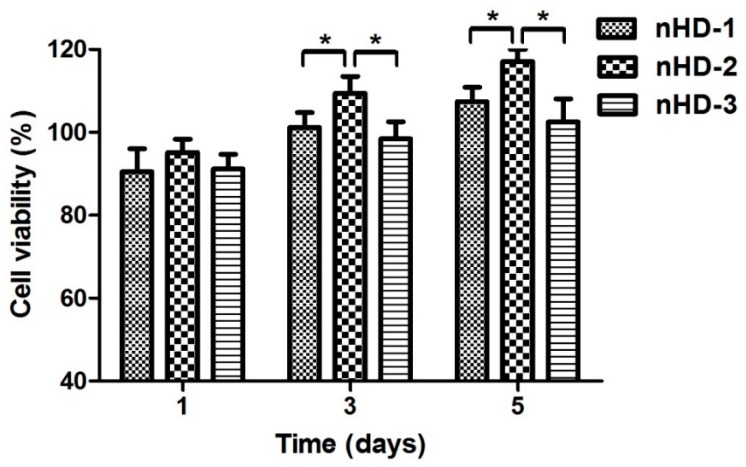
The osteoblast MC3T3-E1 cell viabilities of three nHD scaffolds’ extract solutions after 1, 3, and 5 days of incubation; * *p* < 0.05.

In tissue engineering, three-dimensional biodegradable scaffolds play a role in the extracellular matrix by providing a microenvironment that enhances cell survival; guides cell adhesion and proliferation; enhances extracellular matrix secretion; and targets tissue reconstruction [39,40]. Therefore, for tissue engineering scaffolds, good cell compatibility is one of the most basic conditions. A CCK-8 assay was used to assess the cytotoxicity of the nHD scaffold extract solutions toward MC3T3-E1 osteoblast cells. As shown in Figure 6, after 1 day of incubation, the relative growth rates of cells in the nHD-1, nHD-2 and nHD-3 scaffold extract solutions were 90.5% ± 5.5%, 95.2% ± 3.2% and 91.2% ± 3.6%, respectively. The density of the MC3T3-E1 cells cultured with the extract solutions from the three nHD scaffolds was increased after 3 and 5 days compared to that observed after 1 day. After 5 days of culture, the relative growth rates of cells in the nHD-1, nHD-2 and nHD-3 scaffold extracts were 107.4% ± 3.5%, 117.1% ± 2.9% and 102.5% ± 5.6%, respectively. Compared with that of cells in the nHD-1 and nHD-3 scaffold extracts, the cell viability of cells in the nHD-2 scaffold extract was obviously increased after culturing cells for 1, 3 and 5 days. This was mainly because both the sediment of nano-hydroxyapatite (nHA) from nHD-1 scaffolds with the flabby cross-linking network and the release of residual crosslinking agent in nHD-3 scaffolds had an adverse effect on cell growth. In light of ISO standards (ISO 10993.12-2005), the toxicities of the nHD-1, nHD-2 and nHD-3 scaffolds were all elevated to grade 0, which implied that they all showed good cell compatibility.

### 3.5. Cell Adhesion and Live Cell Staining Analysis

**Figure 7:**
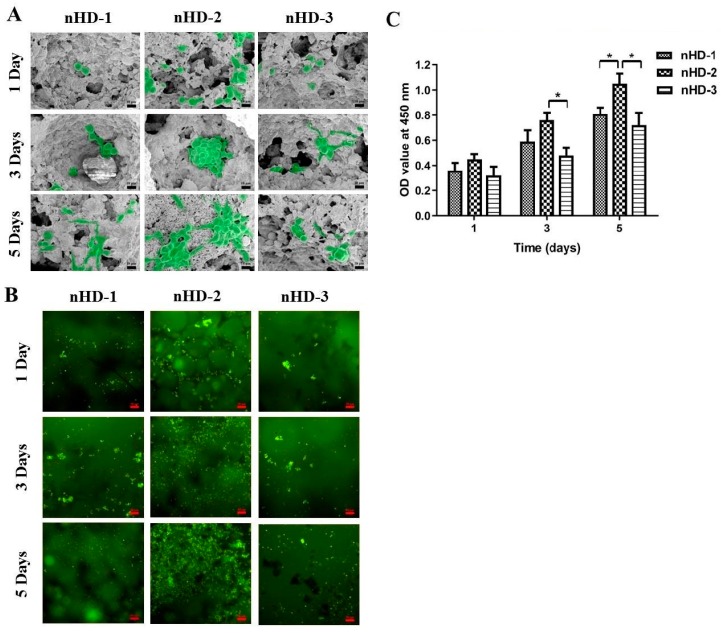
(**A**) The SEM micrographs of MC3T3-E1 cell adhesion on three nHD scaffolds after incubation for 1, 3 and 5 days; the scale bar is 10 μm. (**B**) MC3T3-E1 cells were seeded to three nHD scaffolds and AO staining was done after 1, 3 and 5 days; the scale bar is 50 μm. (C) MC3T3-E1 cells were adhered on three nHD scaffolds after incubation for 1, 3 and 5 days. The OD values at 450 nm were analyzed by the CCK-8 assay; * *p* < 0.05.

The rates of new bone tissue formation are highly influenced by the primal cell adhesion to the scaffolds [31]. To investigate the growth of MC3T3-E1 osteoblast cells on nHD scaffolds, we performed cell adhesion and AO staining experiments. The cell morphologies of MC3T3-E1 cells attached to nHD-1, nHD-2 and nHD-3 scaffolds after incubation for 1, 3 and 5 days were observed by SEM. As depicted in Figure 7A, at 1 day incubation, a large number of MC3T3-E1 cells were attached to the nHD-2 scaffolds, while attached cells on the nHD-1, and nHD-3 scaffolds were scarce. After 3 and 5 days of incubation, unlike that observed on the nHD-1 and nHD-3 scaffolds, a vast majority of the cells were tightly associated with and formed many cell clusters on the nHD-2 scaffolds. Cell adhesion is affected by the scaffold characteristics, such as the porosity, surface topography and surface multicavities [41]. In our study, the high porosity and appropriate pore size of nHD-2 scaffolds can provide larger surface area for cells to infiltrate inside so that they can continue migrating and proliferating. Meanwhile, nHD-2 scaffolds have no adverse effects on MC3T3-E1 cell growth.

In addition, live cell staining analysis was used for further exploration of cell proliferation on scaffolds. Figure 7B indicates that the surfaces of the nHD-2 scaffolds exhibited many more green fluorescence-labeled living cells than the nHD-1 and nHD-3 scaffolds after 1 day of incubation. Along with an increase in the number of days of incubation, the number of living cells on the scaffolds was also gradually increased. On days 3 and 5, a large number of living cells were attached to the nHD-2 scaffolds, while those attached on the nHD-1 and nHD-3 scaffolds were few. This was consistent with the results of the SEM observation on cell adhesion analysis. Moreover, to analyze MC3T3-E1 cell adhesion on nHD scaffolds in a quantitative manner, we used the CCK-8 assay. As shown in Figure 7C, the OD values at 450 nm of the nHD-1, nHD-2 and nHD-3 scaffolds were all enhanced as cell incubation time increased from 1 to 5 days, revealing viable cells throughout the whole culture process with an increase in cell proliferation on the three scaffolds. After 5 days of incubation, the OD values of the nHD-1, nHD-2 and nHD-3 scaffolds were 0.81 ± 0.06, 1.05 ± 0.08 and 0.72 ± 0.1, respectively. Compared with that of the nHD-1 and nHD-3 scaffolds, the OD value of the nHD-2 scaffolds was obviously increased after cultivation for 5 days. Collectively, the above results indicated that the nHD-2 scaffolds significantly stimulated the growth of MC3T3-E1 cells, demonstrating their potential applications as scaffolds for the repair of bone defects for specific purposes, such as the repair of defective bone or repair with mediated materials.

### 3.6. Characteristics of nHD Scaffolds in New Zealand Rabbits

The good histocompatibility and low degradation of the scaffold materials in vivo are vitally important for bone tissue engineering [13,42]. The biocompatibility of each scaffold is affected by the composition, the physicochemical properties and the morphology. In an experiment involving subcutaneous implantation in New Zealand rabbits, not only was the histocompatibility of scaffold materials investigated, but the degradation rates of scaffold materials in vivo were also preliminarily explored.

**Figure 8:**
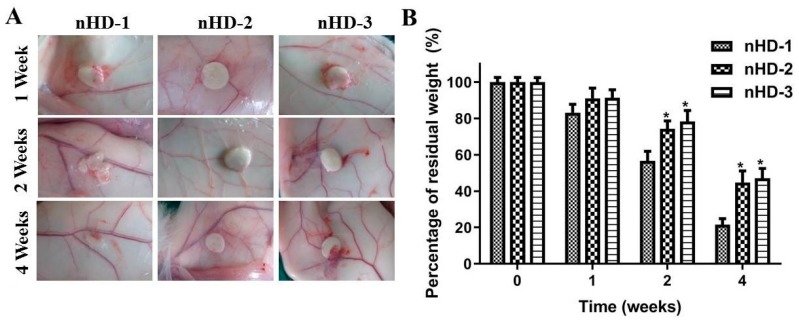
(**A**) The morphologies of tissues around the nHD-1, nHD-2 and nHD-3 scaffolds after 1, 2 and 4 weeks of subcutaneous implantation in New Zealand rabbits. (**B**) The percentage of residual weight for three nHD scaffolds after implantation in New Zealand rabbits at 1, 2 and 4 weeks; * *p* < 0.05 compared with nHD-1 scaffolds.

When the scaffold material is implanted into the rabbit, a series of interactions occur between the blood and the material, resulting in changes in the microvascular system. The assessment of changes in the microvasculature are one way to evaluate the inflammatory reaction [43]. Approximately 1 cm in diameter nHD-1, nHD-2 and nHD-3 scaffolds were implanted subcutaneously into the backs of New Zealand rabbits. As shown in Figure 8A, redness and swelling of the peripheral skin surrounding the nHD-3 scaffolds were observed after 1 week. In contrast, both the nHD-1 and nHD-2 scaffolds had no adverse inflammatory effects on the peripheral skin. After 2 weeks, the nHD-1 scaffolds experienced partial degradation, and the peripheral skin of the nHD-3 scaffolds still exhibited some degree of an inflammatory response, while the nHD-2 scaffolds exhibited almost no degradation or inflammation. At 4 weeks, the nHD-1 scaffolds had degraded to a greater extent compared with the nHD-2 and nHD-3 scaffolds. However, there was still an inflammatory response in the skin surrounding the nHD-3 scaffolds. Furthermore, changes in the masses of the degraded scaffolds after 1, 2 and 4 weeks of subcutaneous implantation were observed separately. As depicted in Figure 8B, the residual weight percentages of the nHD-1, nHD-2 and nHD-3 scaffolds in New Zealand rabbits after 4 weeks of implantation were 21.5% ± 3.4%, 44.8% ± 6.3% and 47.1% ± 5.4%, respectively. As bone tissue regeneration is a long process, the scaffolds with a slower degradation should be matched to the regeneration process [19]. In our study, nHD-2 scaffolds and nHD-3 scaffolds showed the more prolonged endurance after 1, 2 and 4 weeks of subcutaneous implantation. The results were in agreement with the enzymatic degradation assay in vitro results above. Collectively, the above data suggest that nHD-2 scaffolds had better biodegradability and histocompatibility for use in tissue engineering compared to the nHD-1 and nHD-3 scaffolds.

### 3.7. Examination of Repaired Bone Defects

**Figure 9:**
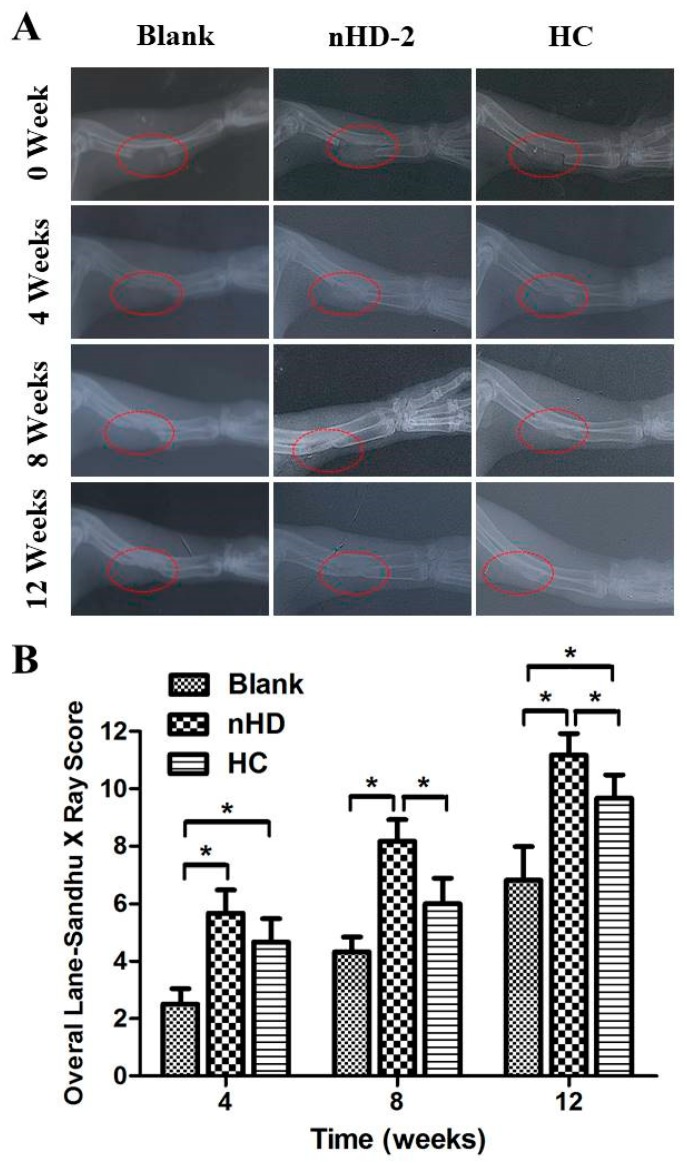
(**A**) The X-ray images of radius defect site after 4, 8 and 12 weeks of nHD-2 scaffolds’ and hydroxyapatite/collagen composite (HC) scaffolds’ repairs in New Zealand rabbits, respectively. Blank groups represent radius defect sites where no scaffolds were implanted into, which were used for comparison with test groups nHD-2 and positive control HC (commercially available bone scaffolds). (**B**) The overall Lane–Sandhu X-ray scores were elevated on the blank group, nHD-2 and HC scaffolds, respectively. * *p* < 0.05.

The ability of bone repair in vivo is essential characteristic of bone substitutes [13]. To evaluate the bone repair effect of the materials, we performed radius defect experiments on rabbits. Under mild sedation, the animals were X-rayed in the prone position with the forelimb stretched so that part of the radius defect could be viewed clearly. Figure 9A shows that 17 mm radii were cut, and gaps were formed; the implanted material comprising the nHD-2 scaffolds and positive control group (HC scaffolds) can also be seen. The two edges of the osteotomy were sharp and clean. After 4 weeks, the edges became blunt. New bone formed during the degradation of implant materials. The formation of new bone accounted for 25%–50% of bone defects in the HC and nHD-2 groups, while the formation of new bone accounted for less than 25% in the blank group. At the end of 8 weeks, in the HC and nHD-2 groups, newly formed bone had interconnected the two edges according to the radiographs. The gaps were filled with new bone in the nHD-2 group, and a small gap still existed in the HC group. Some new bone had formed, and the bone gaps were narrowed slightly in the blank group. At 12 weeks, the HC and nHD-2 scaffolds were both completely degraded. The bone defects had been repaired well by the nHD-2 scaffolds, while in the HC group, the defect was still present. The newly formed bone tissues filled in the entire defective area in the nHD-2 group; in contrast, the HC group showed a slightly decreased volume of newly formed bone tissue. The results showed that new bone formed gradually when nHD or HC degraded. nHD and HC scaffolds could lead to bone regeneration, and the degradation products participated in new bone formation. 

According to the national standard for the Lane–Sandhu X-ray score (Table 1), we then determined the Lane–Sandhu X-ray score for the blank control and the nHD-2 and HC scaffolds. As shown in Figure 9B, after 12 weeks, the overall Lane–Sandhu X-ray scores of the blank control group, nHD-2 scaffold and HC scaffold were 6.83 ± 1.16, 11.17 ± 0.75 and 9.67 ± 0.81, respectively, which demonstrated that the nHD-2 and HC scaffolds presented significant superiority in terms of repairing the defect. Furthermore, compared with the HC scaffolds, the nHD-2 scaffolds presented significantly superior effects in repairing the defect. The results were to a large extent owing to the advantages of excellent biocompatibility of HLC and fine osteoconductivity of n-HA in nHD-2 scaffolds. Thus, leading to a tissue response which is called osteoconduction; it enhances bone bonding to the scaffolds and further promotes the new bone formation [13]. Taken together, these results suggest that nHD-2 scaffolds have a pronounced capacity for bone regeneration and the reconstruction of rabbit radius defects.

### 3.8. H&E Staining

**Figure 10:**
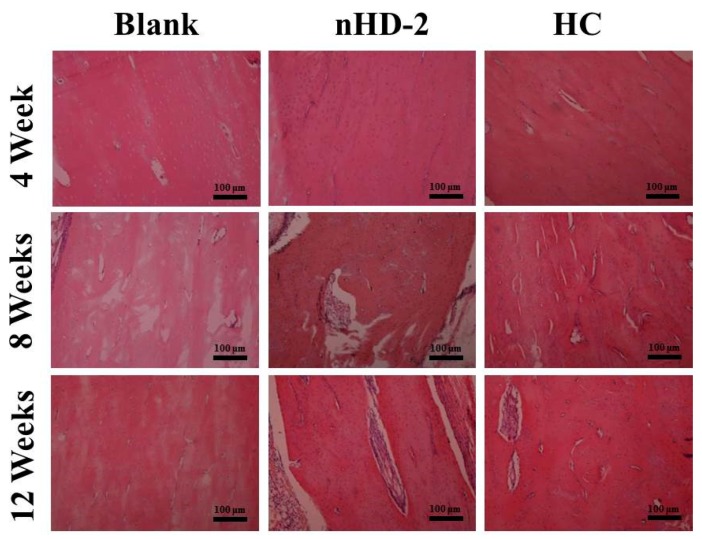
Images of H&E staining in New Zealand rabbits tissue surrounding the radius defect site after 4, 8 and 12 weeks of nHD-2 scaffolds’ and HC scaffolds’ repairs, respectively. The scale bar is 100 μm.

H&E staining is the most widely used morphological observation method in histopathology [44]. The histological responses in the bone repair site at 4, 8 and 12 weeks were investigated via H&E staining in rabbits (Figure 10). After 4 weeks of bone repair, the bone repair sites in the nHD-2 scaffold group and the HC scaffold group showed formation of bone matrix and osteoblasts, while there was little of this in the control group. After 12 weeks of bone repair, the number of bone matrices and osteoblasts in the nHD-2 group and HC group were both increased compared with those in the control group. Moreover, compared to those in the HC group, the bone repair sites in the nHD-2 group exhibited increased amounts of bone matrix and increased numbers of osteoblasts, which demonstrated that nHD-2 scaffolds exhibited better performance in bone regeneration in the rabbit radius defect.

## 4. Conclusions

In summary, we have developed a novel three-dimensional porous nHD scaffold composite by crosslinking with various concentrations of DEO. Compared to nHD-1 scaffolds, nHD-2 and nHD-3 scaffolds were shown to possess superior mechanical strength and resistance to enzymatic hydrolysis due to their higher crosslinking density and more stable network structure. In vitro cell experiments and in vivo subcutaneous injection experiments with these scaffolds demonstrated that nHD-2 scaffolds presented better cytocompatibility and histocompatibility than nHD-1 and nHD-3 scaffolds as a result of the highly interconnected structure and the low level of residual crosslinking agent in nHD-2 scaffolds. Additionally, the bone defect repair performance in vivo indicated that nHD-2 scaffolds exhibited improved guidance during bone regeneration compared to that of commercially obtained HC bone scaffolds. Thus, these results demonstrated that nHD-2 scaffolds might serve as promising candidates for application in bone tissue reconstruction engineering.

## Figures and Tables

**Table 1:** The International Lane–Sandhu X-ray score standard.

Type	Criteria	Score
the bone formation	no bone formation	0
	25% bone formation of the bone defect site	1
	50% bone formation of the bone defect site	2
	75% bone formation of the bone defect site	3
	bone formation filled bone defect site	4
the bone connection	fracture line is clear	0
	part of the fracture line still exists	2
	the fracture line disappears	4
the bone modeling	no bone remodeling	0
	The formation of bone marrow cavity	2
	the remodeling of cortical bone	4
